# Oomycete Diversity and Ecology in Declining Alder Stands in Switzerland

**DOI:** 10.1007/s00248-025-02553-w

**Published:** 2025-05-22

**Authors:** Goda Mizeriene, Vaidotas Lygis, Simone Prospero

**Affiliations:** 1 Swiss Federal Research Institute WSL, Zuercherstrasse 111, CH-8903 Birmensdorf, Switzerland; 2 State Scientific Research Institute Nature Research Centre, Akademijos Str. 2, LT-08412 Vilnius, Lithuania

**Keywords:** *Alnus*, Bark lesions, Rhizosphere soil, Water, Isolation

## Abstract

In this study, we assessed the occurrence and diversity of four oomycete genera (*Phytophthora*, *Phytopythium*, *Pythium*, and *Globisporangium*) in 13 declining alder (*Alnus glutinosa* and *A. incana*) stands in Switzerland. For this, we sampled and analyzed soil from tree rhizosphere, water from streams and rivers along which the stands were located, and symptomatic alder bark. The overall isolation rate was 47.2%, with a total of 400 oomycete isolates recovered at all 13 sites. The highest incidence of oomycete isolates was in soil samples (baiting, 82.5% isolation rate), followed by water (baiting, 14.7%), and bark (direct isolation, 2.7%). Of all recovered oomycete isolates, 90.3% could be successfully assigned to a known species, for a total of 23 species identified, including both preferential saprotrophs and pathogens. Among all genera, *Phytophthora* was the most abundant with 273 isolates (75.6%), followed by *Phytopythium*, *Pythium*, and *Globisporangium*. Oomycete species diversity showed a significant variation among substrates. Only one species—*Phytophthora lacustris*—was abundant in all substrates, while 16 species were restricted to a specific substrate, mainly soil. The rhizosphere of symptomatic alder trees harbored the most diverse oomycete community, highlighting once again the importance of soil as a reservoir for these microorganisms. Only two *Phytophthora* species were isolated from alder bark lesions, namely, *P.* × *alni*, the known causal agent of alder decline, and *P. lacustris*. The low recovery rate of *P.* × *alni* might be due to attempts to isolate it from old, inactive lesions, but may also suggest that alder decline might be caused by other oomycetes infecting the root system of the trees.

**Supplementary Information:**

The online version contains supplementary material available at 10.1007/s00248-025-02553-w.

## Introduction

Oomycetes are filamentous, fungus-like microbial eukaryotes [1]. Thanks to their diverse lifestyles, pathogenicity, and host range, these microorganisms can be considered one of the most successful groups of eukaryotes, which is globally distributed in almost all ecosystems on Earth [2]. Although oomycetes are functionally heterogeneous [3], many of them are severe pathogens of agricultural crops and forest trees [4, 5], with a significant impact on the global food security [6]. Regarding tree health, the genera *Phytophthora* (*P.*), *Pythium* (*Py.*), and *Phytopythium* (*Pp.*) are particularly relevant.

The genus *Phytophthora* includes more than 200 currently known species [7], many of them being plant pathogens [8] and representing a phytosanitary threat to forest ecosystems worldwide [9]. *Pythium* species are not only plant but also animal, algal, or fungal pathogens, as well as saprophytes. Currently, this genus counts more than 140 species, occurring in a wide range of both aquatic and terrestrial habitats [10]. Over the years, the genus *Pythium* has been reclassified, and more recently, based on the phylogeny and the morphology of the sporangium, it has been subdivided into the four genera *Ovatisporangium*, *Globisporangium*, *Elongisporangium*, and *Pilasporangium* [11]. The genus *Phytopythium* is relatively new and was previously assigned to *Pythium* sp. clade K [12]. Uzuhashi et al. [11] reclassified this genus as *Ovatisporangium*, and now *Ovatisporangium* and *Phytopythium* are considered synonyms [13]*.* This genus consists of 26 species [14] found in various habitats [15], mainly as necrotrophic generalists [1].

The introduction rate of pathogenic oomycetes into Europe’s natural ecosystems has increased dramatically over the last few decades [16] and continues to rise. Since the early 1990 s, a severe decline of alder (*Alnus* spp.) has been observed first in Britain and then across the entire continent [17]. The causal agent of this decline was found to be *Phytophthora alni *sensu lato, a previously unknown *Phytophthora* species [18]. Later, it was shown that *P. alni* s. l*.* consists of three species, namely the hybrid *P.* × *alni* and its two parental species *P.* × *multiformis* and *P. uniformis* [19]. These three species differ in morphology, genetic background, virulence, and geographic distribution [20].

In Switzerland, alder dieback has been observed since the early 2000 s, and *P. alni* s. l. was first officially isolated in 2008 from declining alders along the Reuss river [21]. In 2020, the isolates were re-analyzed and definitively attributed to *P.* × *alni* [22]. However, since then, no comprehensive study has been carried out to investigate the diversity of *Phytophthora* species in declining alder stands, although black (*Alnus glutinosa*) and grey (*A. incana*) alders are ecologically important species in riparian ecosystems such as river and stream banks, and lake shores. Recently, Schoebel et al. [23] presented results of a study that investigated *Phytophthora* community composition in Swiss watercourses over the period 2012–2016. A total of 11 *Phytophthora* species were detected, but no conclusions could be drawn linking the *Phytophthora* species found to declining alders.

In the present study, we aimed at answering the following two questions: (i) What is the incidence and diversity of the oomycete species belonging to the genera *Phytophthora*, *Pythium*, *Phytopythium* (*Ovatisporangium*), and *Globisporangium* in declining alder stands in Switzerland? (ii) How do incidence and diversity of the isolated oomycetes vary across sampling sites and substrates (i.e., symptomatic bark, rhizosphere soil, and water)?

## Materials and Methods

### Study Sites

**Fig. 1:**
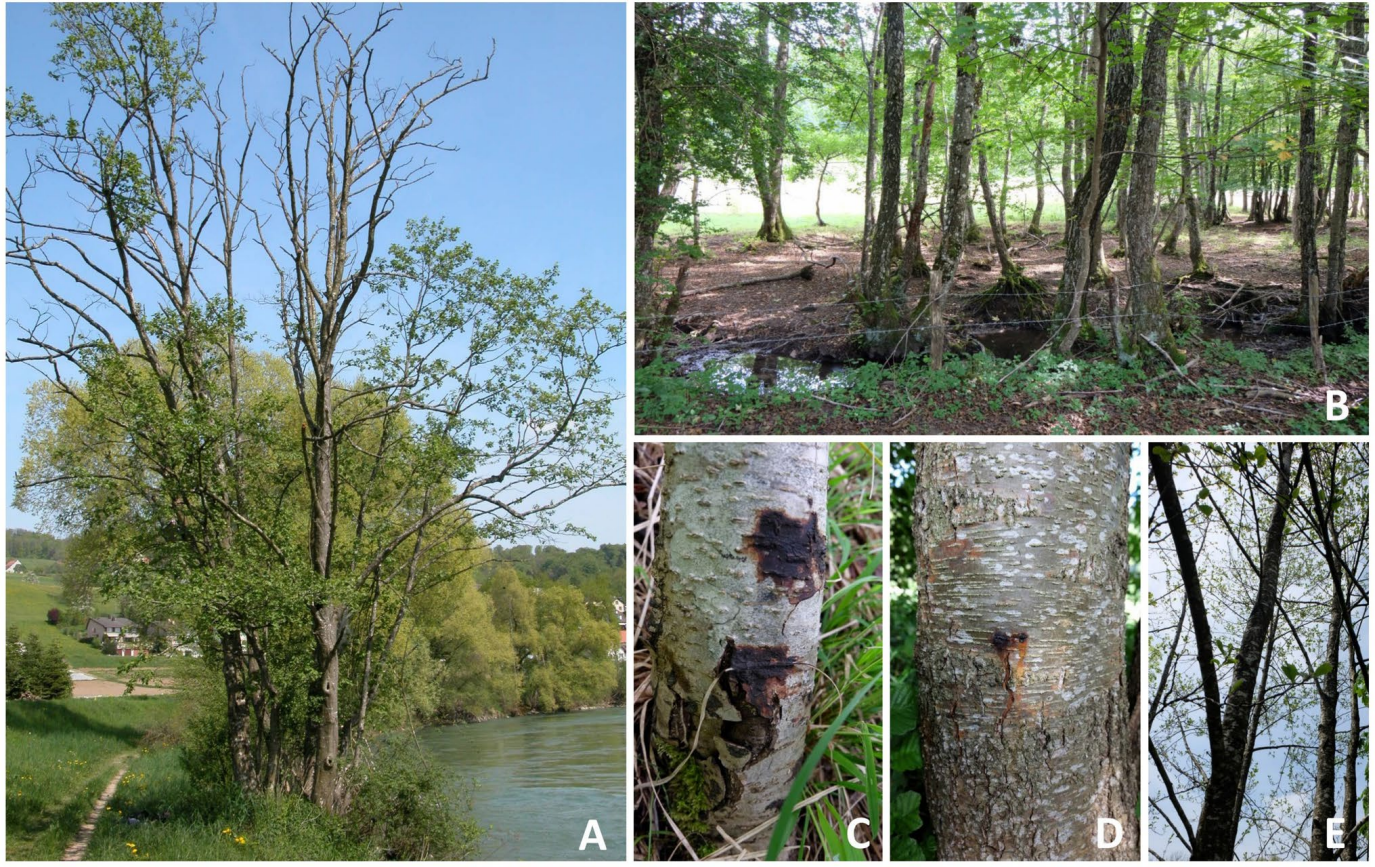
Typologies of sampling sites and disease symptoms. **A** Riparian stand along a river; **B** alder stand along a stream in a marsh; **C** old bleeding lesions on a stem of grey alder (*Alnus incana*); **D** fresh bleeding lesion (tarry spot) on a stem of black alder (*Alnus glutinosa*); **E** grey alders showing crown dieback symptoms (sparse crowns, small leaves) (A:© Swiss Forest Protection, WSL; B-E: © Phytopathology, WSL)

Field sampling was conducted at 13 sites in Switzerland between April and September 2015 (Table 1). The sites were selected with the help of the local forest service at locations (196–840 m a.s.l.) where declining alders (*Alnus incana*, *A. glutinosa*) had been observed. They either consisted of mostly disturbed (management, cattle, hiking) stands along a watercourse (stream or river) or within a marshland (for examples, see Fig. 1A and B). All sites were sampled only once, except for Rottenschwil and Orvin, which were sampled twice in different months (July and September) because of the presence of numerous declining trees. At each site, alder trees with symptoms of decline, such as bark lesions (bleeding cankers, tarry spots on the outer bark) along the stem or at the root collar, and crown thinning (Fig. 1C–E, [18]), were selected for sampling.

**Table 1:** Information about the 13 sites sampled in this study and the number of samples collected

Site	Coordinates (WGS84)		Waterbody name (type)	Sampled *Alnus* species	Forest type	Samples (*N*)		
	Longitude	Latitude				Bark	Soil	Water
Eggenwil	8.3384	47.36432	Reuss (river)	*A. incana*	Riparian	7	7	0
Lauerz	8.58025	47.03799	Chlausenbach (stream)	*A. glutinosa*, *A. incana*	Riparian	8	8	1
Le Landeron	7.05001	47.0499	Vieille Thielle (duct)	*A. glutinosa*	Riparian	1	1	1
Lignières	7.07807	47.09019	Unnamed (pond)	*A. glutinosa*	Riparian	4	4	1
Magadino	8.8694	46.15581	Ticino (river)	*A. incana*	Marsh	6	6	1
Nussbaumen	8.82237	47.61628	Nussbommersee (lake)	*A. incana*	Marsh	2	2	0
Oberglatt	8.51323	47.48062	Glatt (river)	*A. incana*	Marsh	2	2	1
Orvin	7.20246	47.15602	L’Orvine (river)	*A. glutinosa*, *A. incana*	Riparian	11	11	2
Rottenschwil	8.373	47.32264	Reuss (river)	*A. glutinosa*, *A. incana*	Riparian	10	10	2
Schwyzerbrugg	8.7123	47.15264	Biber (river)	*A. incana*	Riparian	0	0	1
Steinerberg	8.57789	47.04523	Goldbach (stream)	*A. incana*	Riparian	2	2	1
Wil	8.51377	47.61742	Schwarzbach (stream)	*A. glutinosa*, *A. incana*	Riparian	2	2	1
Würenlos	8.37022	47.43536	Limmat (river)	*A. glutinosa*	Riparian	1	1	1
Total						56	56	13

A total of 56 alder trees (42 *A. incana* and 14 *A. glutinosa*) were sampled (bark lesions and rhizosphere), ranging from 1 (Le Landeron and Würenlos) to 11 (Orvin) trees per site (Table 1). In Schwyzerbrugg, no trees were sampled because it was too difficult to access them. Thirty-six of the sampled alder trees (64.3%) had bleeding bark lesions on the main stem or at the root collar (11 *A. glutinosa* and 25 *A. incana* trees), 11 trees exhibited a thin crown with decline symptoms (one *A. glutinosa* and ten *A. incana* trees), and nine trees showed both crown decline and bleeding bark lesions (two *A. glutinosa* and seven *A. incana* trees). A total of 13 water samples were collected at 11 sites (two sites, Orvin and Rottenschwil, with two water samples).

### Oomycete Sampling and Isolation

After removing the bark at the margin of a stem lesion with a sterile knife, from one single lesion 20–30 pieces of phloem tissue were taken using a sterilized Jamshidi needle (2 mm in diameter) and placed on a selective CARP+ medium [24] directly in the field. The Petri plates were then incubated in the laboratory at 22 °C in the dark and for 2 to 14 days examined for the presence of oomycete-like hyphae growing out of the samples. When such hyphae were observed, an agar plug from the margin of the colony was transferred with a sterile toothpick onto a new Petri plate containing carrot piece agar (CPA) medium [25] and incubated at 22 °C in the dark to obtain a pure culture.

Soil sampling was performed following the protocol of Tedersoo et al. [26]. Briefly, soil (in total about 1 kg per tree) was sampled along the four cardinal points around a symptomatic tree at 0.5–1.0 m distance from the root collar and at a depth of 5–20 cm using a spade. In the laboratory, soil was sieved through a sieve (mesh size 2 mm) and kept cool until further processing. If a waterbody (river, lake, or pond) with banks covered with *A. glutinosa* or *A. incana* trees was present at the sampling site, approximately 1.5 l of water with sediments has also been collected from that waterbody.

The presence of oomycetes in the collected soil and water samples was assessed using the baiting method proposed by Werres et al. [27]. Briefly, 3 days after sampling at the latest, 200 ml of the sampled soil was put in a plastic container (15 cm × 15 cm × 4 cm) and covered with 400 ml of distilled water. Thereafter, four young, healthy rhododendron leaves were put on the water surface as baits. Similarly, 400 ml of water with sediments has been poured into a plastic container and baited with four young, healthy rhododendron leaves. The containers were then incubated for 16-h light period at 20 °C and 8-h dark period at 15 °C for maximum 10 days. For the isolation of oomycetes, five tissue samples (ca 3 mm × 3 mm in size) were cut from each symptomatic leaf (surface-sterilized) around the formed necrotic spots and placed on Petri plates with CPA medium. The plates were incubated at 22 °C in the dark and pure cultures were obtained as described above for phloem tissue samples [27].

### DNA Extraction

To obtain pure mycelium for better DNA extraction, an agar plug removed from the margin of a growing pure culture was transferred to a new Petri plate containing liquid V8 medium [28]. The plates were then incubated for 5–7 days at 22 °C in the dark until they were ca. 80% covered by the culture. The mycelium was harvested on a filter paper by filtration with a Büchner funnel and a vacuum pump. The collected mycelium was washed off the filter paper with sterile distilled water and placed in a freezer at − 20 °C until the DNA extraction. After lyophilization, DNA was extracted using the DNeasy® Plant Mini kit or the DNeasy® 96 Plant Kit (Qiagen, 96 Hilden, Germany).

### PCR and Sequencing

Isolates were identified to species by sequencing part of the ribosomal ITS region. PCR amplification was conducted in a 20-µl reaction volume containing final concentrations of 2 × Master mix (JumpStart™ RedTaq® Ready Mix (Sigma-Aldrich, Germany)), 0.625 µM of each ITS6 [29] and ITS4 [30] primers, and 1 µl template DNA. The target region was amplified by PCR using a Veriti™ Thermal Cycler (Applied Biosystems, Foster City, CA, USA). Amplification was performed with initial denaturation at 95 °C for 2 min, followed by 35 cycles of denaturation: 95 °C for 30 s, annealing: 55 °C for 30 s, extension: 72 °C for 2 min, and final extension: 72 °C for 10 min. The PCR products were purified using Illustra™ ExoProStar™ PCR and Sequence Reaction Clean-Up Kit (Sigma-Aldrich, Germany), according to the manufacturer’s instructions. For sequencing, BigDye™ Terminator v. 3.1 Cycle Sequencing Kit and BigDye™ x-Terminator Purification Kit (Applied Biosystems, Carlsbad, CA) were used according to manufacturer’s protocol. The sequencing was performed on an ABI 3130 or ABI 3730xl capillary sequencer (Applied Biosystems, USA). The obtained sequences were assembled and edited using the software CLC Main Workbench version 8.0 Beta 4 (Qiagen, Bioinformatics, Denmark). For species identification, sequences (~ 800 bp) were compared with publicly available sequences in the National Center for Biotechnology Information (NCBI; https://blast.ncbi.nlm.nih.gov/Blast.cgi) database with BLAST algorithm. Two sequences were considered to belong to the same species if they showed at least 99% similarity. Representative sequences of all identified species are available in the NCBI database (https://www.ncbi.nlm.nih.gov/) under the accession numbers PV082471 to PV082524 (for details, see Table S1).

### Assessment of Oomycete Diversity

Individual-based rarefaction of Hill numbers (D) [31] was used to estimate oomycete diversity across various sites and substrates. The diversity indicators are characterized by the order *q*, which determines the sensitivity of the index to rare or abundant species. The diversity order *q* = 0 (^0^D) represents species richness and shows the diversity of all species. The diversity order of *q* = 1 (^1^D) displays “typical” species and functions as Shannon Diversity. Finally, the diversity order of *q* = 2 (^2^D) represents Simpson diversity and shows the diversity of dominant species [32]. Interpolated/extrapolated curves were produced with the iNEXT package (v.3.0.0) [33] in RStudio v. 4.2.3 (RStudio PBC, Boston, USA). To investigate whether uneven sample sizes from different sites affect total oomycete species diversity, we produced interpolated/extrapolated curves using iNEXT based on sample coverage (hereinafter SC) data. The iNEXT package *estimateD* function was used to calculate the diversity estimates for the minimum sample size between sites, by excluding uninformative sites. To determine whether diversity differences between sites were statistically significant, the 95% confidence intervals were compared. Since visually comparing all confidence intervals was difficult, the significance of the differences in species diversity in water and soil among sites was also determined by calculating and comparing Shannon diversity indices between sites using Hutcheson’s *t*-test [34].

## Results

### Oomycete Incidence

A total of 400 oomycete (*Phytophthora*, *Pythium*, *Phytopythium*, and *Globisporangium*) isolates were recovered from the three different substrates (bark, soil, water). The overall isolation rate was 47.2%, with oomycetes recovered at all 13 sites. The highest incidence of oomycetes was obtained from soil samples (330 isolates, 82.5% isolation rate), followed by water samples (59 isolates, 14.7% isolation rate), and bark samples (11 isolates, 2.7% isolation rate). Out of the 400 oomycete isolates, 361 (90.3%) could be successfully assigned to a known species, for a total of 23 identified species (Table 2). The remaining 39 isolates could not be unequivocally assigned to a specific oomycete species and were thus removed from further analyses. Among all genera, *Phytophthora* was the most abundant with 273 isolates (75.6%), followed by *Phytopythium* (79 isolates), *Pythium* (six isolates), and *Globisporangium* (three isolates) (Table 2).

**Table 2:** Incidence of identified (i.e., assigned to known species) oomycete taxa recovered from three substrate types (soil, water and bark) sampled in declining alder (*Alnus glutinosa* and *A. incana*) stands in Switzerland (for more information see Materials and Methods section)

Taxa	Soil		Water		Bark	
	Sites^a^	Isolates^b^	Sites	Isolates	Sites	Isolates
*P. citrophthora*	0	-^c^	1	3 (5.80)	0	-
*P. plurivora*	6	113 (53.80)	4	23 (44.20)	0	-
*P. pseudosyringae*	1	1 (0.48)	0	-	0	-
*P. bilorbang*	1	3 (1.43)	0	-	0	-
*P. chlamydospora*	1	2 (0.95)	0	-	0	-
*P. gonapodyides*	4	16 (7.60)	2	2 (3.80)	0	-
*P. heteromorpha*	2	2 (0.95)	0	-	0	-
*P. lacustris*	9	59 (28.10)	6	23 (44.25)	1	4 (36.40)
*P*. × *alni*	1	1 (0.48)	0	-	1	7 (63.60)
*P. niederhauseri*	1	2 (0.95)	0	-	0	-
*P. pseudocryptogea*	1	1 (0.48)	0	-	0	-
*P. honggalleglyana*	1	6 (2.86)	0	-	0	-
*P. gallica*	1	4 (1.90)	1	1 (1.90)	0	-
Genus* Phytophthora*	12	210 (100)	9	52 (100)	2	11 (100)
*Pp. citrinum*	5	19 (25.00)	0	-	0	-
*Pp. litorale*	8	45 (59.21)	1	2 (66.67)	0	-
*Pp. montanum*	1	1 (1.32)	0	-	0	-
*Pp. chamaehyphon*	1	2 (2.63)	1	1 (33.33)	0	-
*Pp. paucipapillatum*	2	3 (3.95)	0	-	0	-
*Pp. vexans*	3	6 (7.89)	0	-	0	-
Genus* Phytopythium*	8	76 (100)	1	3 (100)	0	-
*Py. aquatile*	2	5 (83.33)	0	-	0	-
*Py. lutarium*	1	1 (16.67)	0	-	0	-
Genus *Pythium*	2	6 (100)	0	-	0	-
*G. heterothallicum*	1	1 (33.33)	0	-	0	-
*G. intermedium*	1	2 (66.67)	0	-	0	-
Genus *Globisporangium*	1	3 (100)	0	-	0	-
Total (all genera)	12	295	9	55	2	11

^a^Number of study sites, from which respective oomycete taxon has been isolated

^b^Number of the recovered isolates and their frequency of occurrence within a specific genus (%, shown in brackets)

^c^-, non-applicable

### Oomycete Diversity Across Sampling Sites

**Fig. 2:**
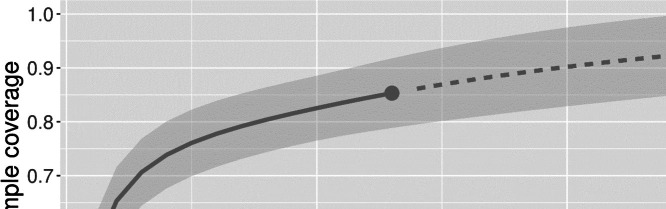
Oomycete sample completeness (sample coverage) based on the sampling design adopted in this study. The solid line represents sample coverage based on rarefaction, while the dashed line is based on extrapolation. The shading in grey indicates the 95% confidence intervals

The highest oomycete diversity was observed in Eggenwil (ten species), Lauerz (nine species), Magadino (eight species), and Orvin and Rottenschwil (seven species each) (Table 3). On the other hand, the sites Le Landeron, Schwyzerbrugg, Steinerberg, Lignières, and Oberglatt exhibited the lowest oomycete diversity with only one to two species isolated from each site (Table 3). Based on the incidence data generated from sampling units (i.e., same sample size across sites), the coverage-based rarefaction and extrapolation sampling curve indicates that the sampling effort in this study was sufficient to catch up to 85% of the expected oomycete diversity (Fig. 2). As stated above, Eggenwil had the highest observed species diversity, but based on rarefied and extrapolated estimates for 22 isolates, Magadino had the highest species richness (^0^D, 8.19), as well as the highest estimations for the most common (together with Eggenwil: ^1^D, 7.49 and 7.53, respectively) and most abundant (^2^D, 7.10) species (Table 3). Seven of the 13 sites sampled had SC scores greater than 0.90, which allows a reliable estimation of oomycete diversity (Table 3).

**Table 3:** Observed and estimated diversity of identified (i.e., assigned to known species) oomycetes recovered from three substrate types (symptomatic bark, rhizosphere soil, and water) in declining alder (*Alnus glutinosa* and *A. incana*) stands in Switzerland (13 sampling sites; for more information see Table 1)

Observed species diversity			Estimated species diversity^a^					
Sampling site	Number of isolates	Number of species	Method^b^	SC^c^	Order *Q*^d^	Diversity estimate of order *q*	*q*D (lower CL)^e^	*q*D (upper CL)^f^
Eggenwil	41	10	R	0.81	0	7.53	6.03	9.03
					1	4.12	2.86	5.38
					2	2.63	1.73	3.54
Lauerz	78	9	R	0.92	0	5.26	4.24	6.29
					1	3.72	3.00	4.44
					2	3.00	2.36	3.63
Le Landeron	8	1	n.a.^g^	n.a	n.a	n.a	n.a	n.a
Lignières	27	2	R	0.99	0	1.97	1.44	2.50
					1	1.29	0.94	1.65
					2	1.16	0.90	1.42
Magadino	20	8	E	0.91	0	8.19	6.25	10.12
					1	7.49	5.70	9.28
					2	7.10	5.11	9.08
Nussbaumen	20	4	E	1.00	0	4.00	3.01	4.99
					1	3.43	2.76	4.11
					2	3.21	2.52	3.89
Oberglatt	9	2	n.a	n.a	n.a	n.a	n.a	n.a
Orvin	49	7	R	0.91	0	5.30	3.99	6.61
					1	3.94	3.11	4.77
					2	3.37	2.73	4.01
Rottenschwil	56	7	R	0.93	0	5.42	4.42	6.42
					1	3.87	2.94	4.79
					2	3.11	2.23	3.98
Schwyzerbrugg	2	2	n.a	n.a	n.a	n.a	n.a	n.a
Steinerberg	5	2	n.a	n.a	n.a	n.a	n.a	n.a
Wil	35	4	R	0.97	0	3.63	3.01	4.25
					1	2.72	2.20	3.23
					2	2.27	1.72	2.82
Würenlos	11	4	E	1.00	0	4.22	1.40	7.04
					1	3.62	1.75	5.49
					2	2.94	1.08	4.80

^a^Calculated for a sample size of *N* = 22 isolates

^b^*R* rarefaction, *E* extrapolation

^c^Estimated sample coverage

^d^Sensitivity of the index to rare or abundant species

^e^The bootstrap lower confidence limits for expected richness (value of 0.95)

^f^The bootstrap upper confidence limits for expected richness (value of 0.95)

^g^*n.a.* not applicable

#### *Phytophthora*

**Fig. 3:**
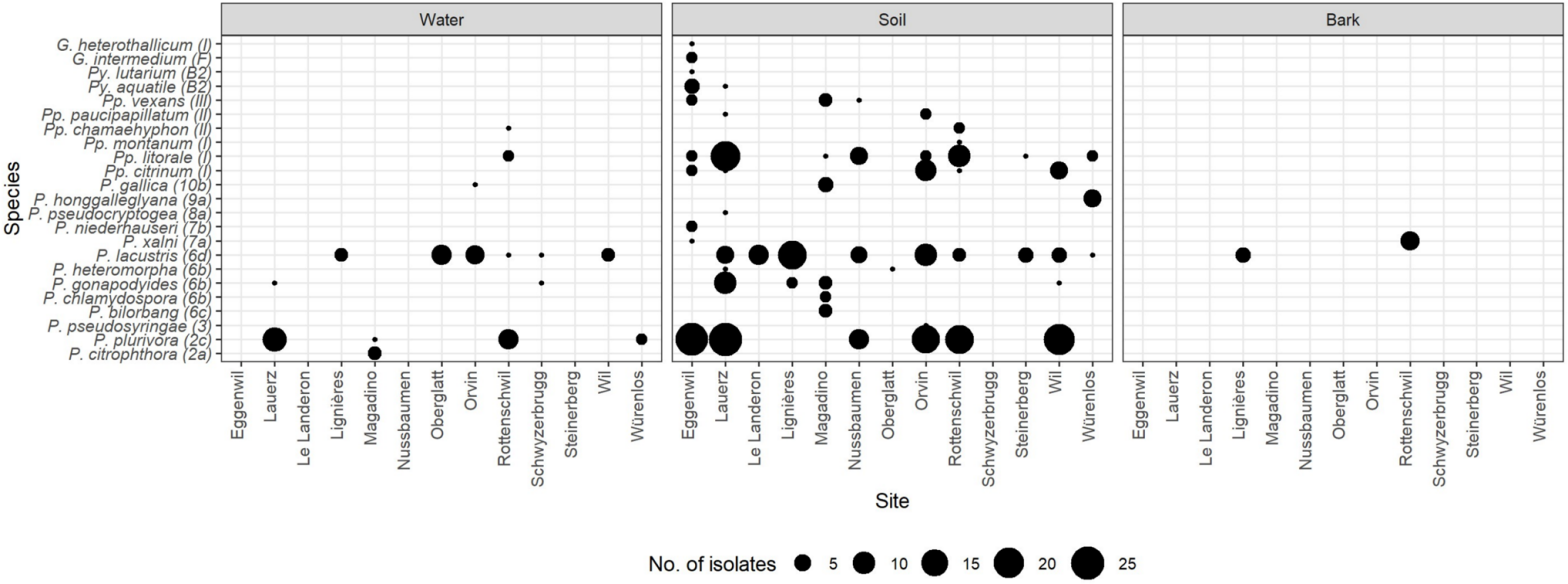
Diversity of identified (i.e., assigned to known species) oomycetes recovered in Switzerland from water samples, rhizosphere soil samples, and bark lesions on symptomatic black alder (*Alnus glutinosa*) and grey alder (*A. incana*) trees. Numbers and letters in the parentheses after species names indicate the clade or group [7, 11, 13, 15] to which each species belongs. For full species names, please see Table 2

*Phytophthora* isolates were recovered at all 13 sites and belonged to 13 different species spanning seven different ITS clades (Fig. 3; [7]). On average, three *Phytophthora* species were detected in each site. The highest diversity was observed at Magadino with a total of six species, most of which belonged to the ITS clade 6 (Fig. 3). Twelve out of 13 detected *Phytophthora* species (all except *P. citrophthora*) were recovered from soil samples, five from water (including *P. citrophthora*), and two from bark lesions (*P.* × *alni* and *P. lacustris*).

#### *Phytopythium*

The genus *Phytopythium* was found at nine of the 13 sites and included seven known species (Table 2, Fig. 3). Rottenschwil showed the highest *Phytopythium* species diversity, with a total of four different species detected. All six detected *Phytopythium* species were recovered from soil samples, two from water, and none from bark lesions.

#### *Pythium* and *Globisporangium*

Representatives of genera *Pythium* and *Globisporangium* were rarely detected at the sampled sites and were isolated exclusively from soil samples (Fig. 3).

### Oomycete Diversity Across Different Substrate Types

Oomycete species diversity and incidence showed a significant variation within and among substrates (Fig. 3). Only one species—*P. lacustris*—was present and abundant in all substrates, while 16 species were restricted to a specific substrate, mainly soil.

**Fig. 4:**
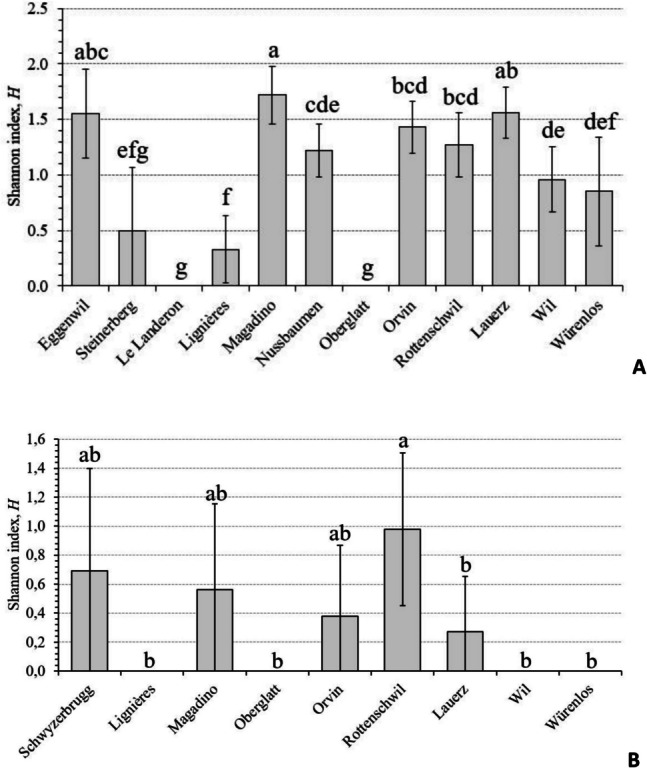
Comparison of Shannon diversity indices among oomycete communities recovered from the rhizosphere soil of symptomatic alder trees (**A**) and water samples (**B**) taken in declining alder stands at 12 (soil) and 9 (water) positive sites (for more information, see Tables 1 and 3). Different letters above the bars indicate statistically significant differences at *p* < 0.05 based on a Hutcheson’s *t*-test. Error bars indicate values of confidence intervals

The rhizosphere of symptomatic alder trees harbored the most diverse oomycete community, with a total of 22 known species (Table 2, Fig. 3). However, most of these species were rather rare and each was represented by less than ten isolates. Typical species present in the rhizosphere of declining alder trees were *P. plurivora*, *P. lacustris*, *Pp. litorale*, and *Pp. citrinum* (altogether they included 80.0% of the identified oomycete isolates recovered from soil). Based on 95% confidence intervals, significant differences were observed among some sites in oomycete diversity in the soil (Fig. S1). These differences were confirmed by a Hutcheson’s *t*-test comparing Shannon diversity indices (Fig. 4A).

The aquatic oomycete community included a total of seven known species (five *Phytophthora* and two *Phytopythium* species) (Table 2, Fig. 3). The most common species in the water samples were *P. lacustris* and *P. plurivora* (23 isolates each; altogether they comprised 83.6% of the identified oomycete isolates recovered from water). Although the overall incidence of both species was the same, the first was found at six sites whereas the latter only at four sites. Notably, *P. citrophthora* was found only in water samples. Four *Phytophthora* species (*P. lacustris*, *P. gallica*, *P. gonapodyides*, and *P. plurivora*) and two *Phytopythium* species (*Pp. chamaehyphon* and *Pp. litorale*) were identified in both soil and water samples. The differences among sites in oomycete species diversity in water were not as marked as in the soil (Fig. S2, Fig. 4B), but the number of water samples analyzed was considerably lower than that of soil samples.

Finally, only two *Phytophthora* species were isolated from alder bark lesions, namely, *P.* × *alni*, the causal agent of alder decline, and *P. lacustris* (Fig. 3).

## Discussion

### Oomycete Abundance and Diversity

Oomycetes were found at all 13 sites sampled, with *Phytophthora* being the most abundant genus followed by *Phytopythium*. The other two investigated genera, *Pythium* and *Globisporangium*, were clearly less frequent. Among the three substrates analyzed, soil yielded the highest number of isolates and species, highlighting once again its importance as a reservoir for these microorganisms. Previous surveys of terrestrial and aquatic oomycete communities occasionally showed higher species diversity in water bodies compared to tree rhizosphere or forest soils (e.g., [35–37]). According to Català et al. [35], water bodies, and rivers in particular, seem to concentrate the inoculum of oomycetes of large areas, especially after rainy periods when the inoculum is discharged into larger bodies of water as runoff. On the other hand, the success of oomycete recovery from one or another substrate may depend on other factors, including the detection technique applied (e.g., [38]).

We found a higher oomycete species diversity, in particular of *Phytophthora* species, in the rhizosphere of declining alder stands compared to other studies carried out in Austria [39], Italy [40], Poland [41], and Turkey [42]. Bregant et al. [43] isolated a similar number (12) of *Phytophthora* species from the rhizosphere of declining *A. glutinosa* trees in Portugal, but only five of them were common to our study. Riit et al. [44] used a metagenomic approach for detection of oomycetes in the rhizosphere soil of declining alder trees across the Fennoscandian and Baltic countries and detected DNA of ten *Phytophthora* species and 28 *Pythium* species. These differences could be partially due to detection methods applied, but also to the influence of abiotic and biotic factors like sampling season, geographic location, and vegetation type (e.g., [45, 46]).

The overall diversity of *Phytophthora* species in our water samples was similar to that reported in surveys conducted in other European countries (e.g., [39, 47, 48]), and in Australia [49]. Of the other oomycete genera, only two species of *Phytopythium* were isolated at low frequencies from water samples during the present study. This is quite surprising as most taxa of the families *Pythiaceae* and *Peronosporaceae* are dependent on aquatic environments and are usually abundantly recovered from natural water bodies (e.g., [48, 50, 51]). This low success of oomycete recovering from water samples may be explained (inter alia) by the fact that we used an ex situ baiting method, while an in situ baiting would probably have allowed capturing more species [38].

Bark lesions on *Alnus* species yielded the lowest number and diversity of oomycetes—we were able to isolate only two *Phytophthora* species. A lower incidence and diversity of *Phytophthora* species in the alder bark than in the soil was also reported in other studies (e.g., [52]) and may be due to several factors, including a suboptimal isolation method (in our case, direct plating of bark samples in the field) or occasional sampling of old lesions in which *Phytophthora* might not be active anymore.

### Insights into the Ecology of the Isolated Oomycete Species

#### The Genus *Phytophthora*

*Phytophthora plurivora*, *P. lacustris*, and *P. gonapodyides* were the most abundant species in the rhizosphere soil and water samples, all of which are widespread in natural and agricultural ecosystems. *Phytophthora plurivora* is an aggressive soil-borne plant pathogen with a broad host range and worldwide distribution, often associated with declining forest trees (e.g., [53]). The high incidence of *P. plurivora* in declining alder stands in Switzerland is in agreement with the results of previous studies conducted in Europe (e.g., [41–43, 54, 55]).

Although the clade 6 member *P. lacustris* is usually regarded as a saprotroph or opportunistic plant pathogen, which is common in riparian ecosystems in Europe and North America [56–58], there is a growing concern about its involvement in the etiology of tree diseases. O’Hanlon et al. [59] speculated that this species may be responsible for black alder decline in Northern Ireland. The pathogen was also found infecting this alder species in Portugal [60], as well as causing diseases in several other plant hosts in Europe [57]. In our study, *P. lacustris*, together with *P.* × *alni*, was the only oomycete species isolated from alder bark lesions. Given the predominance of *P. lacustris* in Swiss watercourses [23], its exact role in causing alder decline in Switzerland should be further investigated.

The third most frequently isolated *Phytophthora* species in our study, *P. gonapodyides*, also belongs to ITS clade 6 and has been traditionally regarded as a weak parasite with saprophytic abilities, usually present in aquatic environments [61]. In Europe, this species was already associated with declining broadleaved trees [53]. However, although in North America *P. gonapodyides* is considered an important species involved in the etiology of native alder species dieback [56], its role in alder decline in Europe is still unclear.

All the other ten *Phytophthora* species identified in our study were restricted to one or two sites and mostly present only in soil. Among them, seven species were already known to occur in Switzerland [23], whereas for *P. heteromorpha*, *P. niederhauseri*, and *P. pseudocryptogea*, this is the first report for the country. *Phytophthora heteromorpha* was first described in Italy from riparian habitats and in inoculation experiments proved to be pathogenic on *A. incana* [62]. *Phytophthora niederhauseri* is a highly pathogenic polyphagous species associated with ornamentals, fruit trees, and native plants, distributed worldwide, including Europe [63, 64]. Finally, *P. pseudocryptogea* is a species within the *P. cryptogea* species complex that was officially described in 2015 [65]. In Turkey, *P. pseudocryptogea* was recovered from the rhizosphere of declining oaks [66], whereas in Canada, the species was reported to cause root rot on western white pine (*Pinus monticola*) in seed orchards [67]. In Italy, it was isolated from declining alder trees [68], which indicates its potential to be pathogenic on trees.

#### The Genera* Phytopythium*, *Pythium*, and *Globisporangium*

The rhizosphere soil of declining alders was found to host a diverse assemblage of species of the genera *Phytopythium*, *Pythium*, and *Globisporangium*, which, to our knowledge, had never been previously investigated in natural ecosystems in Switzerland. These genera are known to include numerous plant pathogens mainly of tree seedlings and herbaceous plants [11, 69]. In this study, two *Phytopythium* species, namely *Pp. litorale* and *Pp. citrinum*, were quite commonly isolated from rhizosphere soil of declining alders*.* Derviş et al. [70] speculated that *Pp. litorale* may be the causal agent of the severe decline of oriental plane in Turkey, while Polat et al. [71] associated it to a kiwifruit dieback. In several European countries, the USA, and Vietnam, *Pp. litorale* was found in watercourses [37, 39, 51, 59]. *Phytopythium citrinum* is known as a common inhabitant of aquatic and riparian ecosystems in Europe and North America [47, 51]. Also, it was isolated from the rhizosphere of declining black alder and pedunculate oak trees in Poland [55, 72]. In a recent study by Christova [47], both *Pp. citrinum* and *Pp. litorale* showed moderate to high potential to infect several woody plant species, as well as some perennial and herbaceous plants. For this reason, both organisms were determined as pathogens with a wide host range. The other five *Phytopythium* species detected in this study were less frequent and limited to a few sites. While *Pp. vexans* is widespread worldwide and shows pathogenicity towards economically important woody hosts to which it causes root rot, damping off, crown rot, stem rot, or patch canker (e.g., [64], and references therein), the published information on the ecology of *Pp. montanum*, *Pp. chamaehyphon*, and *Pp. paucipapillatum* is rather scarce and does not refer to *Alnus* species. Similarly, neither of the two *Pythium* species found in the present study, *Py. aquatile* and *Py. lutarium*, and neither of the two *Globisporangium* species, *G. heterothallicum* and *G. intermedium*, were associated with alder previously.

## Conclusions

Confirming previous studies in Europe and North America, our analyses revealed a diverse and abundant community of oomycetes in declining alder stands in Switzerland, in particular in the rhizosphere of symptomatic trees. The species recovered ranged from known saprotrophs or opportunistic plant pathogens to aggressive pathogens. Noteworthy, only two *Phytophthora* species were isolated from bark lesions on alders, namely, *P.* × *alni*, the known causal agent of alder decline, and *P. lacustris*. Although this might be partially explained by the fact that not all sampled bark lesions were still active, it could also suggest that the observed alder decline might be due to pathogens acting only in the root system and/or abiotic stress factors. Future studies are needed to build up understanding of the ecological role of all oomycete species recovered in such ecosystems as well as their possible interactions with alder and a changing environment. Understanding how oomycete communities are assembled in stands (e.g. based on functional traits, [73]) with different health status would also help to further clarify their role in forest ecosystems.

## Supplementary Information

Below is the link to the electronic supplementary material.

Supplementary file1  **Table S1** Oomycete isolates recovered from declining alder (Alnus glutinosa and A. incana) stands in Switzerland with their respective substrate of origin and sequence GenBank accession numbers. Fig. S1 Oomycete diversity in the rhizosphere soil of symptomatic alder trees at 12 different sites sampled during the present study (for more information see Tables 1 and 3) in Switzerland. The comparison is shown by individual abundance-based rarefaction (solid lines) and extrapolation curves (dashed lines). Each panel is based on the first three Hill numbers [32, 33]: ^0^D (left panel), ^1^D (middle panel) and ^2^D (right panel). The color-shaded regions show the 95% confidence intervals. Fig. S2 Oomycete diversity in water samples taken in the declining alder stands at 9 different sites in Switzerland (for more information see Tables 1 and 3). The comparison is shown by individual abundance-based rarefaction (solid lines) and extrapolation curves (dashed lines). Each panel is based on the first three Hill numbers [32, 33]: ^0^D (left panel), ^1^D (middle panel) and ^2^D (right panel). The color-shaded regions show the 95% confidence intervals. (DOCX 195 KB)

## Data Availability

Representative sequences of all identified species are available in the NCBI database (https://www.ncbi.nlm.nih.gov/) under the accession numbers PV082471 to PV082524.
