# Supplementary material for: Oomycete Diversity and Ecology in Declining Alder Stands in Switzerland
Source: Microb Ecol. 2025 May 22;88:49. doi: 10.1007/s00248-025-02553-w (PMC12098200; doi:10.1007/s00248-025-02553-w)
Supplement: Supplementary file 1 [file 248_2025_2553_MOESM1_ESM.docx]

**Supplementary Materials**

**Table S1** Oomycete isolates recovered from declining alder (Alnus glutinosa and A. incana) stands in Switzerland with their respective substrate of origin and sequence GenBank accession numbers.

| Isolate No. | Species | Isolation source | Accession No. |
| --- | --- | --- | --- |
| GN513 | *P. bilorbang* | Soil | PV082471 |
| GN516 | *P. chlamydospora* | Soil | PV082472 |
| GN518 | *P. chlamydospora* | Soil | PV082473 |
| GN509 | *P. citrophthora* | Water | PV082474 |
| GN519 | *P. citrophthora* | Water | PV082475 |
| GN520 | *P. citrophthora* | Water | PV082476 |
| GN131 | *P. pseudocryptogea* | Soil | PV082477 |
| GN515 | *P. gallica* | Soil | PV082478 |
| GN531 | *P. gallica* | Soil | PV082479 |
| GN536 | *P. gallica* | Soil | PV082480 |
| GN88 | *P. gonapodyides* | Water | PV082481 |
| GN152 | *P. gonapodyides* | Soil | PV082482 |
| GN244 | *P. gonapodyides* | Soil | PV082483 |
| GN507 | *P. heteromorpha* | Soil | PV082484 |
| GN485 | *P. honggalleglyana* | Soil | PV082485 |
| GN491 | *P. honggalleglyana* | Soil | PV082486 |
| GN496 | *P. honggalleglyana* | Soil | PV082487 |
| GN95 | *P. lacustris* | Soil | PV082488 |
| GN229 | *P. lacustris* | Soil | PV082489 |
| GN236 | *P. lacustris* | Soil | PV082490 |
| GN10 | *P. niederhauseri* | Soil | PV082491 |
| GN11 | *P. niederhauseri* | Soil | PV082492 |
| GN5 | *P. plurivora* | Soil | PV082493 |
| GN376 | *P. plurivora* | Soil | PV082494 |
| GN484 | *P. plurivora* | Water | PV082495 |
| GN482 | *P. pseudosyringae* | Soil | PV082496 |
| GN528 | *P. bilorbang* | Soil | PV082497 |
| GN531 | *P. bilorbang* | Soil | PV082498 |
| GN19 | *P.* × *alni* | Soil | PV082499 |
| GN210 | *P.* × *alni* | Bark | PV082500 |
| GN211 | *P.* × *alni* | Bark | PV082501 |
| GN20 | *Py. aquatile* | Soil | PV082502 |
| GN57 | *Py. aquatile* | Soil | PV082503 |
| GN106 | *Py. aquatile* | Soil | PV082504 |
| GN59 | *Py. lutarium* | Soil | PV082505 |
| GN73 | *Pp. chamaehyphon* | Water | PV082506 |
| GN171 | *Pp. chamaehyphon* | Soil | PV082507 |
| GN200 | *Pp. chamaehyphon* | Soil | PV082508 |
| GN43 | *Pp. citrinum* | Soil | PV082509 |
| GN405 | *Pp. citrinum* | Soil | PV082510 |
| GN474 | *Pp. citrinum* | Soil | PV082511 |
| GN71 | *Pp. litorale* | Water | PV082512 |
| GN488 | *Pp. litorale* | Soil | PV082513 |
| GN526 | *Pp. litorale* | Soil | PV082514 |
| GN7 | *Pp. montanum* | Soil | PV082515 |
| GN352 | *Pp. paucipapillatum* | Soil | PV082516 |
| GN468 | *Pp. paucipapillatum* | Soil | PV082517 |
| GN476 | *Pp. paucipapillatum* | Soil | PV082518 |
| GN389 | *Pp. vexans* | Soil | PV082519 |
| GN532 | *Pp. vexans* | Soil | PV082520 |
| GN533 | *Pp. vexans* | Soil | PV082521 |
| GN45 | *G. heterothallicum* | Soil | PV082522 |
| GN16 | *G. intermedium* | Soil | PV082523 |
| GN26 | *G. intermedium* | Soil | PV082524 |

**Fig. S1** Oomycete diversity in the rhizosphere soil of symptomatic alder trees at 12 different sites sampled during the present study (for more information see Tables 1 and 3) in Switzerland. The comparison is shown by individual abundance-based rarefaction (solid lines) and extrapolation curves (dashed lines). Each panel is based on the first three Hill numbers [32, 33]: ^0^D (left panel), ^1^D (middle panel) and ^2^D (right panel). The color-shaded regions show the 95% confidence intervals.

**Fig. S2** Oomycete diversity in water samples taken in the declining alder stands at 9 different sites in Switzerland (for more information see Tables 1 and 3). The comparison is shown by individual abundance-based rarefaction (solid lines) and extrapolation curves (dashed lines). Each panel is based on the first three Hill numbers [32, 33]: ^0^D (left panel), ^1^D (middle panel) and ^2^D (right panel). The color-shaded regions show the 95% confidence intervals.
