# Supplementary material for: Oomycete Diversity and Ecology in Declining Alder Stands in Switzerland
Source: Microb Ecol. 2025 May 22;88(1):49. doi: 10.1007/s00248-025-02553-w (PMC12098200; doi:10.1007/s00248-025-02553-w)
Supplement: Supplementary file 1 — Supplementary file1 Table S1 Oomycete isolates recovered from declining alder (Alnus glutinosa and A. incana) stands in Switzerland with their respective substrate of origin and sequence GenBank accession numbers. Fig. S1 Oomycete diversity in the rhizosphere soil of symptomatic alder trees at 12 different sites sampled during the present study (for more information see Tables 1 and 3) in Switzerland. The comparison is shown by individual abundance-based rarefaction (solid lines) and extrapolation curves (dashed lines). Each panel is based on the first three Hill numbers [32, 33]: 0D (left panel), 1D (middle panel) and 2D (right panel). The color-shaded regions show the 95% confidence intervals. Fig. S2 Oomycete diversity in water samples taken in the declining alder stands at 9 different sites in Switzerland (for more information see Tables 1 and 3). The comparison is shown by individual abundance-based rarefaction (solid lines) and extrapolation curves (dashed lines). Each panel is based on the first three Hill numbers [32, 33]: 0D (left panel), 1D (middle panel) and 2D (right panel). The color-shaded regions show the 95% confidence intervals. (DOCX 195 KB) [file 248_2025_2553_MOESM1_ESM.docx]

**Supplementary Materials**

**Table S1** Oomycete isolates recovered from declining alder (Alnus glutinosa and A. incana) stands in Switzerland with their respective substrate of origin and sequence GenBank accession numbers.

| Isolate No. | Species | Isolation source | Accession No. |
| --- | --- | --- | --- |
| GN513 | *P. bilorbang* | Soil | PV082471 |
| GN516 | *P. chlamydospora* | Soil | PV082472 |
| GN518 | *P. chlamydospora* | Soil | PV082473 |
| GN509 | *P. citrophthora* | Water | PV082474 |
| GN519 | *P. citrophthora* | Water | PV082475 |
| GN520 | *P. citrophthora* | Water | PV082476 |
| GN131 | *P. pseudocryptogea* | Soil | PV082477 |
| GN515 | *P. gallica* | Soil | PV082478 |
| GN531 | *P. gallica* | Soil | PV082479 |
| GN536 | *P. gallica* | Soil | PV082480 |
| GN88 | *P. gonapodyides* | Water | PV082481 |
| GN152 | *P. gonapodyides* | Soil | PV082482 |
| GN244 | *P. gonapodyides* | Soil | PV082483 |
| GN507 | *P. heteromorpha* | Soil | PV082484 |
| GN485 | *P. honggalleglyana* | Soil | PV082485 |
| GN491 | *P. honggalleglyana* | Soil | PV082486 |
| GN496 | *P. honggalleglyana* | Soil | PV082487 |
| GN95 | *P. lacustris* | Soil | PV082488 |
| GN229 | *P. lacustris* | Soil | PV082489 |
| GN236 | *P. lacustris* | Soil | PV082490 |
| GN10 | *P. niederhauseri* | Soil | PV082491 |
| GN11 | *P. niederhauseri* | Soil | PV082492 |
| GN5 | *P. plurivora* | Soil | PV082493 |
| GN376 | *P. plurivora* | Soil | PV082494 |
| GN484 | *P. plurivora* | Water | PV082495 |
| GN482 | *P. pseudosyringae* | Soil | PV082496 |
| GN528 | *P. bilorbang* | Soil | PV082497 |
| GN531 | *P. bilorbang* | Soil | PV082498 |
| GN19 | *P.* × *alni* | Soil | PV082499 |
| GN210 | *P.* × *alni* | Bark | PV082500 |
| GN211 | *P.* × *alni* | Bark | PV082501 |
| GN20 | *Py. aquatile* | Soil | PV082502 |
| GN57 | *Py. aquatile* | Soil | PV082503 |
| GN106 | *Py. aquatile* | Soil | PV082504 |
| GN59 | *Py. lutarium* | Soil | PV082505 |
| GN73 | *Pp. chamaehyphon* | Water | PV082506 |
| GN171 | *Pp. chamaehyphon* | Soil | PV082507 |
| GN200 | *Pp. chamaehyphon* | Soil | PV082508 |
| GN43 | *Pp. citrinum* | Soil | PV082509 |
| GN405 | *Pp. citrinum* | Soil | PV082510 |
| GN474 | *Pp. citrinum* | Soil | PV082511 |
| GN71 | *Pp. litorale* | Water | PV082512 |
| GN488 | *Pp. litorale* | Soil | PV082513 |
| GN526 | *Pp. litorale* | Soil | PV082514 |
| GN7 | *Pp. montanum* | Soil | PV082515 |
| GN352 | *Pp. paucipapillatum* | Soil | PV082516 |
| GN468 | *Pp. paucipapillatum* | Soil | PV082517 |
| GN476 | *Pp. paucipapillatum* | Soil | PV082518 |
| GN389 | *Pp. vexans* | Soil | PV082519 |
| GN532 | *Pp. vexans* | Soil | PV082520 |
| GN533 | *Pp. vexans* | Soil | PV082521 |
| GN45 | *G. heterothallicum* | Soil | PV082522 |
| GN16 | *G. intermedium* | Soil | PV082523 |
| GN26 | *G. intermedium* | Soil | PV082524 |


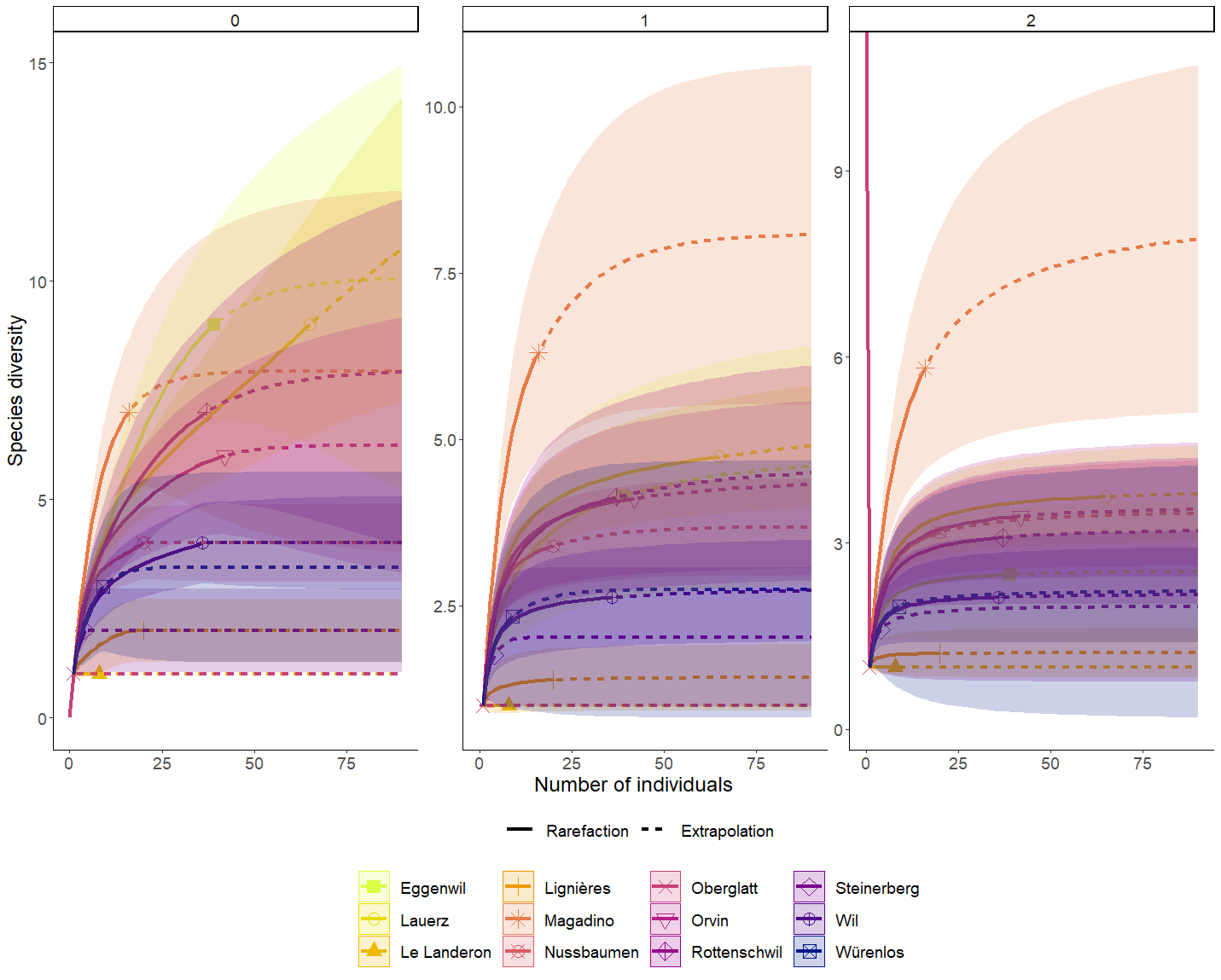


**Fig. S1** Oomycete diversity in the rhizosphere soil of symptomatic alder trees at 12 different sites sampled during the present study (for more information see Tables 1 and 3) in Switzerland. The comparison is shown by individual abundance-based rarefaction (solid lines) and extrapolation curves (dashed lines). Each panel is based on the first three Hill numbers [32, 33]: ^0^D (left panel), ^1^D (middle panel) and ^2^D (right panel). The color-shaded regions show the 95% confidence intervals.


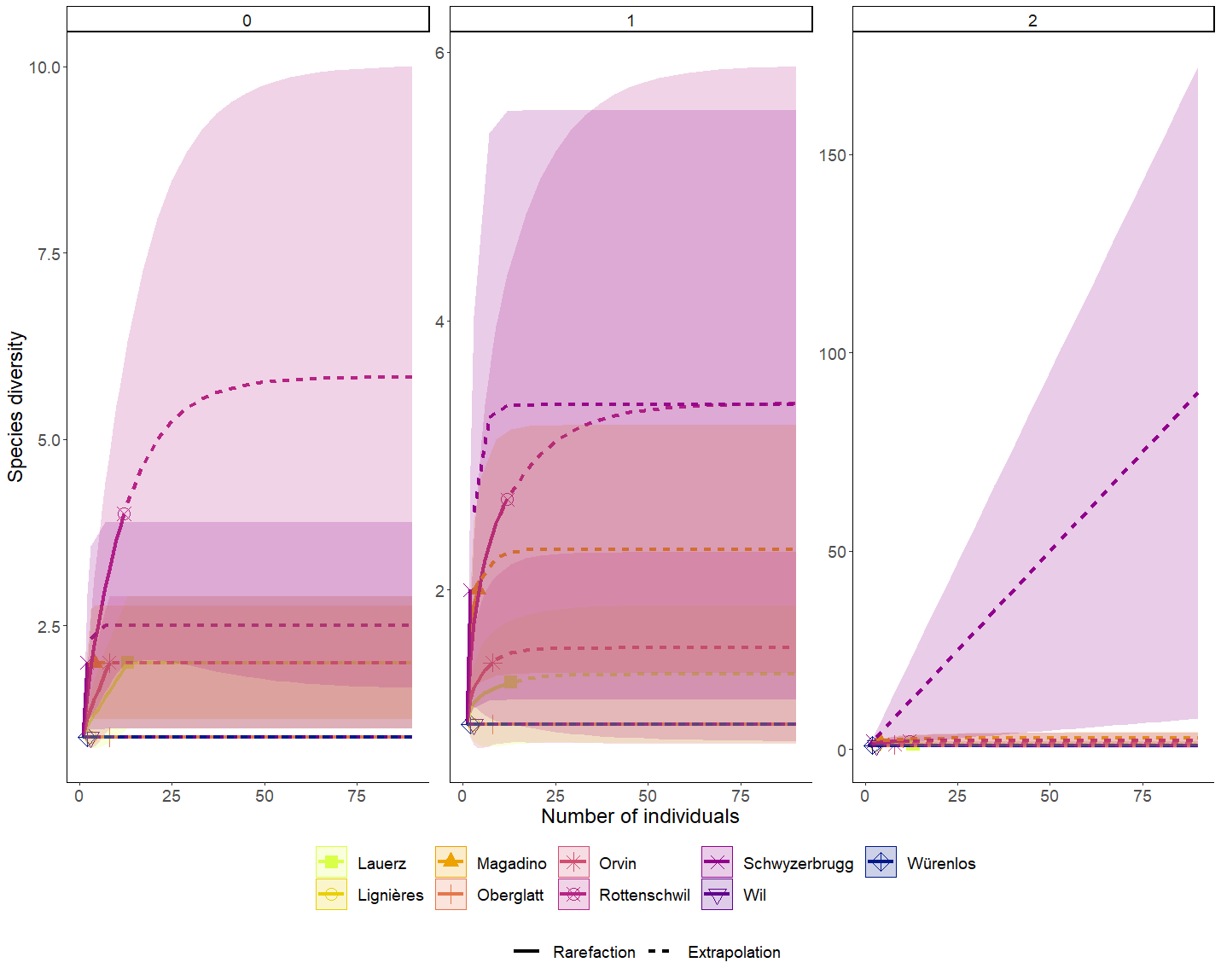


**Fig. S2** Oomycete diversity in water samples taken in the declining alder stands at 9 different sites in Switzerland (for more information see Tables 1 and 3). The comparison is shown by individual abundance-based rarefaction (solid lines) and extrapolation curves (dashed lines). Each panel is based on the first three Hill numbers [32, 33]: ^0^D (left panel), ^1^D (middle panel) and ^2^D (right panel). The color-shaded regions show the 95% confidence intervals.
